# Learning to recover weak signals: A two-stage graph-based framework for all-weather RGB-T object detection

**DOI:** 10.1371/journal.pone.0354466

**Published:** 2026-08-05

**Authors:** Ruoyan Pei, Pengge Ma, Jinwang Qian, Hai Yang, Junling Sun

**Affiliations:** School of Electronics and Information, Zhengzhou University of Aeronautics, Zhengzhou, China; Yanshan University, CHINA

## Abstract

Object detection in complex traffic environments remains challenging due to illumination-induced feature degradation and weak responses from distant small objects. To address these limitations, we propose a two-stage multimodal detection framework that enhances perception under degraded conditions. The first stage employs a Dual-Path Attention Fusion Module (DPAFM) to adaptively integrate RGB and thermal features via learnable gated attention, mitigating modality-specific degradation. The second stage introduces a Hierarchical Attention Graph Neural Network (HA-GNN), which models detection candidates as graph nodes and performs hierarchical relational reasoning over their appearance and spatial relationships to compensate for weakened local cues. Extensive experiments on the KAIST and R-LiViT datasets demonstrate that our framework significantly improves robustness across day and night scenarios, reducing the day-night performance gap from 72.4% to 7.4% and substantially enhancing the detection of small objects. Moreover, the method achieves a real-time inference speed of 27.4 FPS, offering a favorable trade-off between accuracy and efficiency for autonomous driving perception in challenging environments.The code is available at:https://github.com/yangjiepry/YOLO-HA-GNN.git.

## 1. Introduction

Object detection in intelligent transportation systems remains a cornerstone for ensuring safe autonomous driving. While visible-light detectors have achieved remarkable performance [1,2], they often fail in complex traffic environments due to illumination-induced feature collapse, glare, or low-light conditions [3,4]. Incorporating thermal (T) information has emerged as a robust approach because infrared sensors capture heat signatures that are largely invariant to illumination changes [5]. Nevertheless, simple modality fusion does not guarantee reliable detection, as each sensor remains susceptible to specific failure modes in unconstrained traffic scenarios, which are further compounded by remaining difficulties such as modality inconsistency, extreme illumination variations, and adverse weather conditions [6–10].

Extensive efforts have been devoted to pixel-level and feature-level alignment to alleviate the effects of inherent thermal noise and cross-modality feature misalignment [11–13], yet existing RGB-T detection frameworks continue to struggle with the effective fusion of complementary RGB and thermal information, resulting in suboptimal weak signal recovery, particularly for distant or small objects [14–16]. When local discriminative cues are annihilated by sensor degradation or environmental noise [17,18], conventional architectures suffer from high miss rates [19–21]. This deficiency largely arises from the absence of mechanisms for contextual verification: the capacity to assess the plausibility of candidate detections using surrounding spatial and appearance relationships.

Neuroscientific insights into visual processing suggest that robust perception relies on the integration of local and global information [22,23]. In particular, the Gestalt completion principle [24] indicates that human vision reconstructs missing local information by leveraging relational dependencies across the visual field [25,26]. Recent multi-modal learning approaches [27,28] have advanced detection performance by integrating complementary information, but the recovery of weak signals from degraded small objects remains a persistent bottleneck [29]. We argue that such degraded local cues can be effectively compensated by explicitly modeling candidate detections as relational graphs, capturing both appearance and topological dependencies with their surrounding context [30,31].

Motivated by these observations, we propose a two-stage neural pattern verification framework designed to systematically address object pattern degradation. To clarify the main differences from existing RGB-T detection methods, our framework highlights a decoupled two-stage design, an efficient graph construction strategy, and a tailored cross-modal interaction mechanism, demonstrating explicit representation and efficiency advantages over conventional CNN-, global Transformer-, and recent Mamba-based approaches under severe signal degradation. The first stage, the Dual-Path Attention Fusion Module (DPAFM), leverages the proposed cross-modal interaction mechanism with learnable gated attention to adaptively integrate RGB and thermal features, suppressing modality-specific noise and mitigating feature collapse. The second stage introduces the Hierarchical Attention Graph Neural Network (HA-GNN), which implements a targeted graph construction strategy that decouples appearance and spatial attention to perform high-level contextual reasoning, allowing weak or degraded nodes to “borrow” semantic strength from neighboring candidates. This architecture is inspired by the neurocomputational principle that ventral and dorsal visual streams process “what” and “where” information, respectively, enabling robust perception under uncertain conditions.

The key innovations of this work are summarized as follows:

- **Dual-Path Attention Fusion Module (DPAFM):** adaptively fuses RGB and thermal modalities using a well-formulated cross-modal interaction mechanism governed by learnable gated attention to mitigate modality-specific degradation and suppress feature collapse, bypassing the immense computational burden of dense Transformer or Mamba blocks.
- **Hierarchical Attention Graph Neural Network (HA-GNN):** decoupled appearance and spatial attention mechanisms and a dynamic graph construction strategy enable sparse contextual reasoning, allowing weak or missing local features to be compensated through relational dependencies among candidate nodes, providing a clear precision and structural advantage over traditional CNN and global multi-modal approaches.
- **Comprehensive Validation:** extensive evaluations on the KAIST and R-LiViT datasets demonstrate a reduction of the day-night performance gap from 72.4% to 7.4%, a 275% improvement in small-object detection $\left(\text{A}{\text{P}}_{\text{S}}\right)$ and real-time inference at 27.4 FPS, achieving a favorable trade-off between accuracy and efficiency.

Integration of multi-modal fusion, hierarchical relational reasoning, and neuro-inspired attention mechanisms establishes a generalizable paradigm for object detection under extreme environmental conditions, bridging the gap between local feature degradation and reliable contextual verification.

## 2. Related Work

### 2.1. RGB-T Object Detection

Object detection leveraging paired visible (RGB) and thermal (T) modalities has evolved rapidly to alleviate the fundamental limitations of single-modality sensors under unconstrained environments. Early research primarily utilized standard convolutional neural networks (CNNs) to align and fuse cross-modal features at the pixel, feature, or decision level. However, conventional CNN architectures often struggle with spatial misalignment and are highly susceptible to thermal noise under complex background conditions. To overcome these barriers, recent advanced approaches have introduced implicit cross-attention structures and specialized alignment layers to adaptively recalibrate multi-modal feature distributions [32]. Concurrently, state-space models (SSMs) and Mamba-based paradigms have emerged as potent alternatives to global Transformers, offering linear computational complexity while capturing long-range contextual dependencies across different modalities [33]. Distinct from these dense fusion or uniform state-space architectures that process entire scenes indiscriminatingly, our proposed framework establishes a decoupled two-stage paradigm, which employs a sparse graph reasoning strategy to explicitly verify candidate object patterns while suppressing non-target background clutter.

### 2.2. All-weather object detection

Robust object detection under all-weather scenarios represents a critical yet challenging requirement for autonomous driving and intelligent transportation systems. Extreme conditions such as heavy fog, torrential rain, and severe illumination variations drastically degrade the discriminative cues of visible cameras, causing catastrophic feature collapse. To address these adverse conditions, modern detection frameworks generally adopt multi-object architectures that dynamically regulate modality contributions based on ambient environmental illumination and weather attributes [34]. Another mainstream line of research focuses on low-level adverse weather removal or restoration prior to the detection backbone, ensuring the stability of downstream semantic representations [35]. Beyond standard RGB-T perception, recent advances in broader multi-modal spectra—such as hyperspectral imaging—have demonstrated immense capacity in capturing fine-grained physical attributes under severe signal degradation [36]. This has further driven the development of multi-spectral foundation models, such as SpectralGPT, which leverage large-scale pre-training to acquire robust foundational visual priors for thermal and multispectral perception [37]. While these foundation models require substantial computational resources, our framework focuses on local signal recovery and efficient real-time deployment via a targeted dual-path attention fusion mechanism and a hierarchical graph network.

### 2.3. RGB-D/T salient object detection

Salient object detection (SOD) aims to identify the most visually distinctive regions in a scene and serves as a highly correlated dense prediction task that shares core cross-modal interaction challenges with RGB-T object detection. Both fields rely heavily on mining complementary cross-modal information to resolve background clutter, ambiguous object boundaries, and sensor degradation. To effectively capture cross-modal synergies, state-of-the-art RGB-D and RGB-T SOD models have explored multi-scale and multi-level feature fusion topologies to achieve granular information transfer [38]. To handle complex cross-modal variations, researchers have leveraged physical priors to guide cross-modal attention mechanisms [39], as well as hybrid prompt-driven segment architectures to exploit zero-shot capabilities of foundational vision models [40].

Moreover, advanced structured topologies, such as confluent triple-flow configurations [41] and intra-modality self-enhancement mirror networks [42], have been proposed to systematically eliminate cross-modal contradictions. In the domain of RGB-D SOD, modern designs emphasize computational efficiency and progressive reasoning, producing lightweight and efficient network variations [43], adaptive attention regulation frameworks [44], and progressive decoding mechanisms [45]. To capture global context without incurring overwhelming complexity, recent methods utilize multi-scale awareness global fusion [46] alongside perceptual localization and focus refinement strategies [47]. Although these SOD frameworks are optimized for dense pixel-level classification, their core concepts of adaptive modal interaction, progressive noise suppression, and structural enhancement provide strong theoretical inspirations for our two-stage object verification scheme.

### 2.4. Discussion: Differences from Contemporary Architectures

To further clarify the technical uniqueness and architectural merits of our proposed two-stage neural pattern verification framework, we explicitly contrast it with three prevailing vision paradigms:

**• Compared with CNN-based frameworks:** Standard CNNs rely heavily on localized receptive fields and grid-structured convolutions. When facing severe sensor degradation or adverse weather, local object patterns undergo catastrophic feature collapse, leaving CNNs insufficient for weak signal recovery. In contrast, our HA-GNN explicitly constructs a structured candidate relation graph, enabling severely degraded object nodes to globally “borrow” semantic and contextual strength from neighboring robust candidates.

**• Compared with Transformer-based frameworks:** Although global Transformers capture long-range dependencies, they suffer from quadratic computational complexity and are highly prone to incorporating dense background noise in unconstrained traffic scenes. Our framework circumvents this by employing a decoupled two-stage design: the DPAFM adaptively filters out modality-specific noise via gated cross-modal interaction, while the HA-GNN performs sparse relational reasoning exclusively on candidate nodes, achieving a superior trade-off between representation capacity and computational efficiency.

**• Compared with Mamba-based frameworks:** Recent state-space models (SSMs), such as Mamba, achieve linear complexity but fundamentally rely on 1D sequential scanning mechanisms (e.g., bidirectional or multi-directional flattening) to process 2D images. This sequential causal scanning inherently distorts the native, non-causal 2D spatial layout and topological relations among independent objects. Our framework eliminates this limitation by treating candidate objects as discrete graph nodes, naturally preserving the permutation-invariant spatial and appearance relationships essential for robust verification.

## 3. Method

### 3.1. Overall framework overview

**Fig 1:**
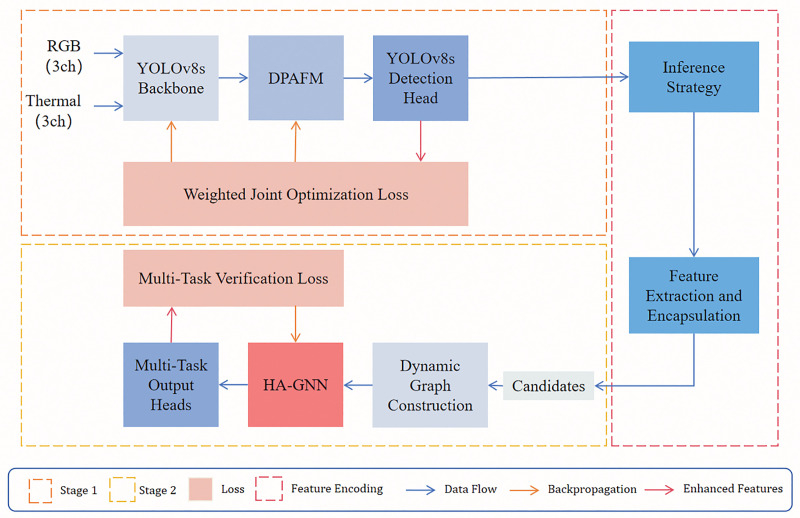
Overall architecture of the proposed algorithm.

The proposed detection framework addresses object pattern degradation through a two-stage “over-complete mining + structured reasoning” paradigm, inspired by dual-process theories in cognitive neuroscience [5]. As illustrated in Fig. 1, the framework decouples detection into two sequential stages with complementary objectives. Stage 1 generates high-coverage candidates from multi-modal RGB-T inputs, prioritizing recall, while Stage 2 verifies and refines these candidates through graph-based contextual reasoning, emphasizing precision. This design shifts recognition from evaluating each candidate independently to context-aware joint optimization, where relationships among candidates provide compensatory evidence for degraded local patterns.

Training proceeds in a step-wise manner to stabilize the optimization of the two stages. Stage 1 focuses on candidate mining, while Stage 2 focuses on relational reasoning and verification. Candidate representations from Stage 1 are structured to facilitate graph-based reasoning in Stage 2, enabling the model to recover weak or missing features by leveraging contextual dependencies.

During inference, Stage 1 generates candidate boxes and corresponding feature representations, which are then passed to the trained Hierarchical Attention Graph Neural Network (HA-GNN). The graph network produces refined detection outputs through hierarchical reasoning, without requiring additional post-processing such as NMS. The two-stage design enforces a clear division of labor: Stage 1 maximizes coverage under challenging conditions, and Stage 2 ensures precise verification, forming a cohesive mining-to-reasoning loop for robust detection under complex traffic scenarios.

### 3.2. Stage 1: High-Recall Multi-Modal Base Detector

The first stage is designed for over-complete mining of all potential object patterns. Even under severe environmental degradation, this stage must retain true-object candidates to provide rich context for subsequent graph-based reasoning. We adopt a “lenient entry, strict exit” strategy, emphasizing pattern coverage completeness and delegating false positive elimination to the second-stage HA-GNN module.

Input images are first processed using a letterbox algorithm, which synchronously scales both RGB and thermal modalities to 1280 × 1280 with gray padding, preserving aspect ratio and geometric integrity. The framework employs a pseudo-siamese two-stream backbone to process the dual modalities:

The equations should be inserted in editable format from the equation editor.

**• RGB branch:** A YOLOv8s CSPDarknet backbone extracts texture and color patterns under sufficient illumination, capturing fine-grained object details.

**• Thermal branch:** A parallel YOLOv8s CSPDarknet extracts illumination-invariant thermal patterns, providing robust structural features in low-light or glare-affected environments.

To effectively integrate multi-modal features, the Dual-Path Attention Fusion Module (DPAFM) is applied after the backbone. DPAFM employs learnable gated attention to adaptively fuse RGB and thermal feature maps, enabling dynamic, illumination-aware compensation of degraded patterns. The fused features are therefore optimized to retain weak signals while suppressing modality-specific noise.

Training of Stage 1 emphasizes recall over precision. Positive samples are assigned using a relaxed matching strategy to maximize candidate coverage, while the total loss combines multiple objectives with weighted contributions:


$$\begin{array}{c} {\text{L}}_{\text{total}}=\lambda_{\text{CIoU}}{\text{L}}_{\text{CIoU}}+\lambda_{\text{DFL}}{\text{L}}_{\text{DFL}}+\lambda_{\text{BCE}}{\text{L}}_{\text{BCE}} \end{array}$$ (1)

where ${\text{L}}_{\text{CIoU}}$, ${\text{L}}_{\text{DFL}}$, and ${\text{L}}_{\text{BCE}}$ correspond to bounding box regression, distribution focal loss, and classification objectives, respectively. The weighting coefficients λ are selected to prioritize spatial localization and small-object preservation.

During inference, Stage 1 outputs candidate boxes along with their multi-scale features from P3, P4, and P5 layers, normalized spatial coordinates, and preliminary confidence scores. These representations are subsequently provided to Stage 2 for hierarchical contextual verification and precise detection.

#### 3.2.1. Dual-path attention fusion module.

**Fig 2:**
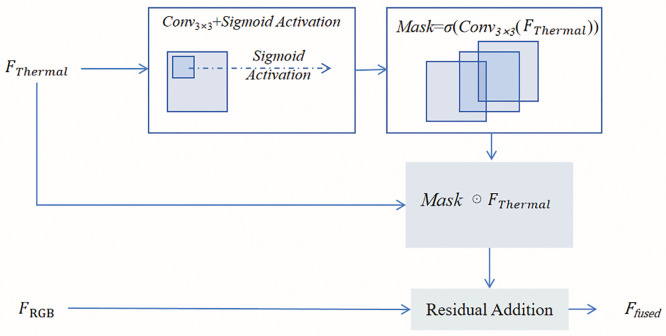
Dual-Path Attention Fusion Module (DPAFM).


The Dual-Path Attention Fusion Module (DPAFM) is embedded at the backbone outputs, namely the P3, P4, and P5 layers before PAN-FPN, as illustrated in Fig 2, It employs a residual gated attention mechanism to adaptively inject reliable thermal patterns into RGB features. Convolutional layers perceive the spatial and channel distribution of thermal features and generate a confidence mask reflecting the importance of thermal patterns:

$$\begin{array}{c} \text{Mask}=\sigma \left(\text{Con}{\text{v}}_{\text{3}\times \text{3}}\left(F_{\text{thermal}}\right)\right) \end{array}$$ (2)

where σ denotes the Sigmoid function, and Conv3 × 3 extracts local contextual information. From the perspective of pattern fusion, Mask can be interpreted as a confidence estimate for reliable pattern regions in the thermal modality. Regions with values closer to 1 indicate more trustworthy thermal patterns.

Meanwhile, a learnable weighting parameter β, initialized to 0, is introduced to achieve a smooth transition from “pure RGB patterns” to “RGB + thermal compensation patterns”:


$$\begin{array}{c} F_{\text{fused}}=F_{\text{rgb}}+\beta \cdot \left(\text{Mask}⊙F_{\text{thermal}}\right) \end{array}$$ (3)

where ⊙ denotes element-wise multiplication.

This design enables the network to dynamically adjust the pattern contribution weights between modalities according to illumination conditions. Under sufficient lighting, β remains small, and the model prioritizes fine-grained texture patterns from RGB images. In low-light environments, β gradually increases, enhancing the injection of thermal radiation patterns to compensate for degraded RGB patterns.

#### 3.2.2. High-Recall Training Strategy.

The first-stage detector is designed to provide an over-complete candidate set for the subsequent graph reasoning module. Therefore, its optimization objective is different from that of a conventional one-stage detector. Instead of directly pursuing highly confident final predictions, the first stage is expected to preserve as many potential object hypotheses as possible, especially for small, distant, occluded, and low-contrast objects. These weak candidates may have low classification confidence in the first stage, but they can still contain useful visual or thermal cues for the second-stage HA-GNN.

To this end, we adopt a high-recall-oriented training and inference strategy. This strategy consists of three key designs: task-aligned positive sample matching, weighted multi-task optimization, and lenient candidate generation during inference. The former two improve the learning quality of candidate localization and semantic discrimination, while the latter prevents weak object responses from being prematurely discarded.

• **Positive Sample Matching:** During training, the Task-Aligned Assigner (TAL) in YOLOv8 is adopted to dynamically assign positive samples. Unlike fixed-threshold matching strategies that rely mainly on IoU, TAL selects positive samples according to a task-aligned criterion that jointly considers classification confidence and localization quality. This mechanism is beneficial for degraded object patterns because candidates with imperfect localization but meaningful semantic responses can still be involved in the training process.

For small or low-light objects, object boundaries are often ambiguous, and local appearance features are easily weakened by noise, occlusion, or insufficient illumination. Under such conditions, overly strict positive sample assignment may discard many difficult yet informative candidates, leading to insufficient supervision for weak object patterns. By using TAL, the first-stage detector can learn from these ambiguous samples and improve its ability to generate high-recall candidate proposals.

• **High-Recall-Oriented Joint Optimization:** The first-stage detector is optimized using a weighted multi-task loss, including bounding box regression loss, distribution focal loss, and classification loss. The total loss is defined as:


$$\begin{array}{c} L_{\text{stage1}}=\lambda_{\text{box}}L_{\text{CIoU}}+\lambda_{\text{dfl}}L_{\text{DFL}}+\lambda_{\text{cls}}L_{\text{BCE}} \end{array}$$ (4)

where ℒ_CIoU_ denotes the bounding box regression loss, ℒ_DFL_ denotes the distribution focal loss for boundary distribution modeling, and ℒ_BCE_ denotes the binary cross-entropy classification loss. The coefficients λ_box_, λ_dfl_, and λ_cls_ are used to balance the contributions of different optimization terms.

In this work, a relatively larger weight is assigned to the bounding box regression term. This design encourages the detector to produce spatially reliable candidate boxes, which is essential for the subsequent graph construction stage. If the candidate locations are inaccurate, the spatial relationships among graph nodes may become unreliable, weakening the effectiveness of graph reasoning. The distribution focal loss further improves boundary quality, while the classification loss maintains category-level discrimination.

This loss design is consistent with the objective of high-recall candidate generation. The first stage is not required to make the final decision for every candidate. Instead, it should provide a sufficiently complete and spatially meaningful proposal set, so that the second-stage HA-GNN can further perform candidate verification, contextual reasoning, and false-positive suppression.

• **Optimizer and Hyperparameter Settings:** The AdamW optimizer is used to train the first-stage detector. Compared with standard stochastic gradient descent, AdamW provides more stable optimization when learning heterogeneous visible and thermal features. A linear learning rate decay strategy is adopted to gradually reduce the learning rate during training, which improves convergence stability in the later optimization stage.

The training and candidate-generation hyperparameters are summarized in Table 1. The loss weights control the relative importance of localization, boundary modeling, and semantic classification during training, while the confidence and NMS thresholds determine the number and diversity of retained candidate proposals during inference.

**Table 1:** Hyperparameter settings for high-recall candidate generation.

Hyperparameter	Value
Box loss weight $\lambda_{\text{box}}$	10.0
DFL loss weight $\lambda_{\text{dfl}}$	1.5
Classification loss weight $\lambda_{\text{cls}}$	1.0
Confidence threshold	0.001
NMS IoU threshold	0.6
Number of GNN neighbors K	10

• **Candidate Generation During Inference:** During candidate generation for the second-stage HA-GNN, a lenient inference strategy is adopted. Specifically, the confidence threshold is set to 0.001, and the non-maximum suppression IoU threshold is set to 0.6, as shown in Table 1

The extremely low confidence threshold is used to retain weak object responses that would normally be removed by conventional detectors. This is particularly important for small objects and low-light objects because their local features are often incomplete, blurred, or low in contrast. Although these candidates may have low first-stage classification confidence, they may still correspond to true objects and can be recovered through contextual reasoning in the second stage.

Meanwhile, the relatively high NMS IoU threshold prevents the detector from prematurely suppressing overlapping proposals. In dense traffic scenes, multiple small objects may appear close to each other, and partially occluded objects may generate highly overlapping candidate boxes. A strict NMS threshold may remove such candidates incorrectly. By retaining more overlapping proposals, the first stage provides a richer candidate graph for HA-GNN.

Although this lenient candidate generation strategy inevitably introduces more false positives, it is suitable for the proposed two-stage framework. The first stage focuses on candidate completeness, while the second-stage HA-GNN is responsible for candidate verification and refinement. Through appearance similarity modeling, spatial-geometric consistency analysis, and hierarchical attention-based message passing, HA-GNN suppresses redundant background proposals and enhances weak object candidates. In our experiments, this strategy preserves more than 85% of potential objects, providing an information-rich foundation for structured graph reasoning.

### 3.3. Candidate box feature extraction and encapsulation

To enable structured reasoning in the second-stage HA-GNN, the candidate boxes generated by the first-stage detector are converted into graph-compatible node representations. Each candidate is described by three types of information: spatial coordinates, multi-scale appearance features, and first-stage prediction scores. These representations provide the necessary node attributes for subsequent graph construction, attention-based message passing, and candidate verification.

For each candidate box ${\text{B}}_{\text{i}}$, RoI Align is applied to the multi-scale feature maps extracted from the backbone, including P3, P4, and P5. These feature levels provide complementary semantic information. Specifically, P3 preserves fine-grained spatial details and is more sensitive to small objects; P4 encodes intermediate local structures that are useful for medium-scale objects; and P5 contains stronger semantic information for large or context-dependent objects. By extracting candidate-level features from all three pyramid levels, the model can obtain a more complete representation across different object scales.

Let ${\text{F}}_{\text{P3}}^{\text{i}}$, ${\text{F}}_{\text{P4}}^{\text{i}}$, and ${\text{F}}_{\text{P5}}^{\text{i}}$ denote the RoI-aligned feature maps of candidate ${\text{B}}_{\text{i}}$ from P3, P4, and P5, respectively. These feature maps are first flattened and then concatenated to form a multi-scale candidate descriptor:


$$\begin{array}{c} F_{\text{cat}}^{i}=\text{Concat}\left(\text{Flatten}\left(F_{P\text{3}}^{i}\right),\text{Flatten}\left(F_{P\text{4}}^{i}\right),\text{Flatten}\left(F_{P\text{5}}^{i}\right)\right) \end{array}$$ (5)

The concatenated feature vector is then passed through two fully connected layers for feature compression and dimensionality reduction:


$$\begin{array}{c} F_{i}=\text{F}{\text{C}}_{\text{2}}\left(\text{ReLU}\left(\text{F}{\text{C}}_{\text{1}}\left(F_{\text{cat}}^{i}\right)\right)\right) \end{array}$$ (6)

where ${\text{F}}_{\text{i}}\in R^{\text{256}}$ denotes the final appearance embedding of candidate ${\text{B}}_{\text{i}}$. In our implementation, the RoI-aligned feature from each pyramid level is resized to 7×7 with 256 channels. Therefore, the concatenated feature vector has a dimension of 7×7×256×3. The first fully connected layer reduces this vector to 1024 dimensions, and the second fully connected layer further projects it to a 256-dimensional candidate embedding.

In addition to the appearance embedding, each candidate retains its normalized spatial coordinates and first-stage prediction scores. The spatial coordinate vector is represented as ${\text{B}}_{\text{i}}=\left[{\text{x}}_{\text{c}},{\text{y}}_{\text{c}},\text{w},\text{h}\right]$, where $\left({\text{x}}_{\text{c}},{\text{y}}_{\text{c}}\right)$ denotes the normalized center location, and (w,h) denotes the normalized width and height. The score vector is denoted as ${\text{S}}_{\text{i}}$ which contains the object confidence score and the category prediction scores generated by the first-stage detector.

After feature extraction and encapsulation, the input to the second-stage HA-GNN consists of three components: candidate boxes, candidate appearance embeddings, and first-stage prediction scores. For a candidate set containing N proposals, the box matrix has the size N×4, the feature matrix has the size N×256, and the score matrix has the size N×(1+C), where C denotes the number of object categories. This structured representation can be written compactly as:


$$\begin{array}{c} C_{\text{graph}}={\left\{\left(B_{i},F_{i},S_{i}\right)\right\}}_{i=\text{1}}^{N} \end{array}$$ (7)

Here, ${\text{B}}_{\text{i}}$ provides the spatial prior for geometric relationship modeling, ${\text{F}}_{\text{i}}$ provides the appearance descriptor for visual similarity computation, and ${\tilde{\text{S}}}_{\text{i}}$ provides the initial confidence prior for candidate verification. These three components jointly define the node attributes used by HA-GNN.

More specifically, the encapsulated candidate representation contains the following information

**• Spatial location information:** The normalized coordinate vector ${\text{B}}_{\text{i}}=\left[{\text{x}}_{\text{c}},{\text{y}}_{\text{c}},\text{w},\text{h}\right]$ describes the position and scale of the candidate box in the image plane. This information is used to construct spatial-geometric relationships among candidate nodes.

**• Visual appearance information:** The 256-dimensional embedding ${\text{F}}_{\text{i}}$ encodes the multi-scale visual characteristics of the candidate. It integrates fine details from P3, local structural cues from P4, and high-level semantic information from P5. This representation allows the appearance attention layer to measure feature similarity between nodes and capture potential co-occurrence relationships.

**• Initial confidence information:** The score vector ${\text{S}}_{\text{i}}$ contains the objectness confidence and class prediction scores from the first-stage detector. These scores serve as prior information for the second-stage verification process. Rather than directly discarding low-confidence candidates, HA-GNN re-evaluates them by considering contextual evidence from neighboring nodes.

Through this candidate encapsulation process, the dense first-stage detection outputs are transformed into structured node representations. This design establishes the interface between the image-level detector and the graph-level reasoning module, enabling HA-GNN to perform candidate refinement based on appearance similarity, spatial consistency, and confidence priors.

### 3.4. Stage 2: Hierarchical Attention Graph Neural Network

After the candidate generation stage, a high-recall candidate set $C={\left\{{\text{B}}_{\text{i}}\right\}}_{\text{i}=\text{1}}^{\text{N}}$ is obtained. The purpose of this stage is to retain as many potential object regions as possible. However, such an over-complete candidate set inevitably contains redundant proposals, ambiguous background patterns, and low-confidence object hypotheses. For each candidate proposal ${\text{B}}_{\text{i}}$, the corresponding visual representation is denoted as ${\text{F}}_{\text{i}}$, and its spatial coordinates are represented as ${\text{B}}_{\text{i}}=\left({\text{x}}_{\text{i}},{\text{y}}_{\text{i}},{\text{w}}_{\text{i}},{\text{h}}_{\text{i}}\right)$, where $\left({\text{x}}_{\text{i}},{\text{y}}_{\text{i}}\right)$ denotes the box center and $\left({\text{w}}_{\text{i}},{\text{h}}_{\text{i}}\right)$ denotes its width and height after normalization.

For small objects and low-light targets, the local appearance feature ${\text{F}}_{\text{i}}$ may be severely degraded due to weak texture, blurred boundaries, low contrast, noise corruption, and insufficient object pixels. Therefore, directly classifying each candidate independently may lead to unstable predictions and a high false-positive rate. To overcome this limitation, the proposed hierarchical attention graph neural network, denoted as HA-GNN, reformulates candidate verification and classification as a joint graph reasoning problem. Instead of relying only on isolated local features, HA-GNN explicitly models the contextual dependencies among candidates by constructing a graph in which candidate proposals are regarded as nodes and their pattern associations are represented as edges.

The key idea of HA-GNN is that object candidates in an image are not independent. They usually exhibit certain visual co-occurrence relationships and spatial layout regularities. By aggregating information from relevant neighboring candidates, weak or degraded local representations can be enhanced through contextual compensation. As illustrated in Fig 1 HA-GNN contains three main components: dynamic graph construction, hierarchical attention-based message passing, and multi-task prediction.

#### 3.4.1. Dynamic graph construction.

Given the candidate set C, HA-GNN first constructs a sparse candidate relation graph G=(V,E), where each node ${\text{v}}_{\text{i}}\in V$ corresponds to a candidate bounding box ${\text{B}}_{\text{i}}$, and each edge ${\text{e}}_{\text{ij}}\in E$ describes the contextual dependency between candidate ${\text{B}}_{\text{i}}$ and candidate ${\text{B}}_{\text{j}}$. The node representation is initialized by jointly encoding the appearance feature and the spatial feature of each candidate.

Specifically, the initial hidden state of node ${\text{v}}_{\text{i}}$ is computed as:


$$\begin{array}{c} {\text{h}}_{i}^{\text{0}}=\delta \left(W_{\text{0}}\left[F_{i}||B_{i}\right]+b_{\text{0}}\right) \end{array}$$ (8)

where ${\text{F}}_{\text{i}}$ denotes the appearance feature extracted from the candidate region, ${\text{B}}_{\text{i}}$ denotes the normalized spatial coordinate vector, [·∥·] represents feature concatenation, ${\text{W}}_{\text{0}}$ and ${\text{b}}_{\text{0}}$ are learnable projection parameters, and δ(·) is a nonlinear activation function. This initialization embeds both visual and geometric information into a unified latent space, which provides the basis for subsequent graph reasoning.

To determine the neighborhood structure of the graph, the pairwise relationship between two candidates is measured from both appearance and geometric perspectives. The appearance similarity between two nodes is calculated by the cosine similarity of their feature embeddings:


$$\begin{array}{c} S_{ij}^{\text{app}}=\frac{F_{i}^{T}F_{j}}{{\left\|F_{i}\right\|}_{\text{2}}{\begin{array}{c} F_{j} \end{array}}_{\text{2}}} \end{array}$$ (9)

Meanwhile, the spatial similarity is defined according to the relative geometric distance between two proposals:


$$\begin{array}{c} S_{ij}^{\text{geo}}=\text{exp}\left(-\frac{{\left\|\begin{array}{c} B_{i}-B_{j} \end{array}\right\|}_{\text{2}}^{\text{2}}}{\sigma^{\text{2}}}\right) \end{array}$$ (10)

where σ controls the sensitivity of the spatial distance measurement. A smaller σ emphasizes nearby candidates, whereas a larger σ allows the model to consider a wider spatial context.


$$\begin{array}{c} \text{Si}{\text{m}}_{ij}=\lambda_{\text{app}}S_{ij}^{\text{app}}+\lambda_{\text{geo}}S_{ij}^{\text{geo}} \end{array}$$ (11)

The final pairwise similarity is obtained by combining appearance similarity and geometric similarity:

where $\lambda_{\text{app}}$ and $\lambda_{\text{geo}}$ are balancing coefficients that control the relative importance of visual consistency and spatial proximity.

For each node ${\text{v}}_{\text{i}}$, the top-K most relevant nodes are selected according to $\text{Si}{\text{m}}_{\text{ij}}$ to form its neighborhood N(i). A directed edge is then established from ${\text{v}}_{\text{i}}$ to each selected neighbor:


$$\begin{array}{c} e_{ij}\in ℰ,\;j\in \text{TopK}\left(\text{Si}{\text{m}}_{ij}\right) \end{array}$$ (12)

This top-K strategy produces a sparse dynamic graph, which reduces computational complexity while preserving the most informative contextual relationships. Compared with a fully connected graph, the proposed dynamic graph construction avoids introducing excessive noisy connections and enables each candidate to aggregate information mainly from semantically or spatially relevant neighbors.

#### 3.4.2. Hierarchical Attention-Based Message Passing.

After constructing the candidate relation graph, HA-GNN performs hierarchical message passing to progressively enhance node representations. The hierarchical design contains three successive reasoning levels: appearance-aware attention, spatial-geometric attention, and recurrent feature fusion. These levels are designed to capture complementary dependencies among candidate proposals.

**• Appearance-Aware Attention:** The first reasoning layer focuses on mining visual co-occurrence relationships among candidates. Since candidates belonging to the same object category or sharing similar object patterns may exhibit correlated visual responses, the appearance-aware attention layer adaptively evaluates the visual relevance between node ${\text{v}}_{\text{i}}$ and its neighbor ${\text{v}}_{\text{j}}$.

For each edge ${\text{e}}_{\text{ij}}$, the unnormalized appearance attention coefficient is calculated as:


$$\begin{array}{c} e_{ij}^{\text{app}}=\text{LeakyReLU}\left(a_{\text{app}}^{T}\left[W_{\text{app}}{\text{h}}_{i}^{\text{0}}∥W_{\text{app}}{\text{h}}_{j}^{\text{0}}\right]\right) \end{array}$$ (13)

where ${\text{W}}_{\text{app}}$ is a learnable feature transformation matrix and ${\text{a}}_{\text{app}}$ is the learnable attention vector. The normalized attention weight is then obtained by applying the softmax operation over the neighborhood of node ${\text{v}}_{\text{i}}$:


$$\begin{array}{c} \alpha_{ij}^{app}=\frac{exp\left(e_{ij}^{app}\right)}{\sum_{k\in N(i)}exp\left(e_{ik}^{app}\right)} \end{array}$$ (14)

The appearance-enhanced representation of node ${\text{v}}_{\text{i}}$ is computed by weighted aggregation of its neighboring node features:


$$\begin{array}{c} h_{i}^{app}=\delta \left(\sum_{j\in Ni}\alpha_{ij}^{app}W_{app}h_{j}^{\text{0}}\right) \end{array}$$ (15)

Through this operation, visually informative neighbors are assigned larger weights, while irrelevant or noisy candidates are suppressed. This is particularly useful when the target candidate itself has weak local texture but its neighboring proposals provide complementary visual evidence.

**• Spatial-Geometric Attention:** Although appearance similarity is useful for capturing visual co-occurrence, it may be unreliable in low-light or cluttered scenes. Therefore, the second reasoning layer introduces spatial-geometric attention to explicitly encode the layout relationships among candidate boxes. This layer aims to model relative position, scale variation, and spatial consistency, which are important cues for distinguishing true object regions from background proposals.

First, the geometric position of candidate ${\text{B}}_{\text{i}}$ is encoded as:


$$\begin{array}{c} \phi \left(B_{i}\right)=\text{PE}\left(x_{i}\right)⊕\text{PE}\left(y_{i}\right)⊕\text{PE}\left(w_{i}\right)⊕\text{PE}\left(h_{i}\right) \end{array}$$ (16)

where PE⋅ denotes positional encoding and ⊕ represents feature concatenation. This encoding maps the normalized box coordinates into a high-dimensional geometric representation, allowing the model to capture fine-grained spatial variations.

For each pair of connected nodes, the geometric attention coefficient is computed by jointly considering the appearance-enhanced node features and the geometric encodings:


$$\begin{array}{c} e_{ij}^{\text{geo}}=a_{\text{geo}}^{T}\left[h_{i}^{\text{app}}\begin{array}{c} h_{j}^{\text{app}} \end{array}\phi \left(B_{i}\right)∥\phi \left(B_{j}\right)\right] \end{array}$$ (17)

where ${\text{a}}_{\text{geo}}$ is a learnable geometric attention vector. The normalized geometric attention weight is defined as:


$$\begin{array}{c} \alpha_{ij}^{geo}=\frac{exp\left(e_{ij}^{geo}\right)}{\sum_{k\in N(i)}exp\left(e_{ik}^{geo}\right)} \end{array}$$ (18)

The spatially enhanced node representation is then obtained as:


$$\begin{array}{c} h_{i}^{geo}=\delta \left(\sum_{j\in N(i)}\alpha_{ij}^{geo}W_{geo}h_{j}^{app}\right) \end{array}$$ (19)

where ${\text{W}}_{\text{geo}}$ is a learnable transformation matrix. By introducing geometric attention, HA-GNN can exploit spatial layout patterns and suppress candidates that are visually similar but spatially inconsistent.

**• Recurrent Feature Update and Fusion:** To further refine the candidate representation, HA-GNN adopts a recurrent update mechanism to perform iterative contextual evolution. At each message passing layer, node ${\text{v}}_{\text{i}}$ receives aggregated messages from its neighborhood and updates its hidden state using a gated recurrent unit. The aggregated message is defined as:


$$\begin{array}{c} m_{i}^{\left(l\right)}=\sum_{j\in N(i)}\alpha_{ij}^{\left(l\right)}W^{\left(l\right)}h_{j}^{\left(l\right)}, \end{array}$$ (20)

where ${\text{h}}_{\text{j}}^{\left(\text{l}\right)}$ denotes the hidden representation of node ${\text{v}}_{\text{j}}$ at the l-th layer, and $\alpha_{\text{ij}}^{\left(\text{l}\right)}$ denotes the corresponding attention weight.

The hidden state is updated as:


$$\begin{array}{c} h_{i}^{\left(l+\text{1}\right)}=\text{GRU}\left(h_{i}^{\left(l\right)},m_{i}^{\left(l\right)}\right),l=\text{0},\text{1},\ldots ,L-\text{1} \end{array}$$ (21)

The GRU-based update controls the amount of newly aggregated contextual information and preserves useful historical information from the previous layer. This gated mechanism prevents over-smoothing and improves the stability of graph reasoning, especially when the candidate graph contains noisy or redundant proposals.

Finally, the initial node feature and the graph-refined feature are fused through a residual transformation:


$$\begin{array}{c} H_{i}=\text{LayerNorm}\left(W_{f}\left[h_{i}^{\text{0}}∥h_{i}^{L}\right]+b_{f}\right) \end{array}$$ (22)

where ${\text{H}}_{\text{i}}$ is the final context-enhanced representation of candidate ${\text{B}}_{\text{i}},{\text{h}}_{\text{i}}^{\text{0}}$ preserves the original candidate information, and ${\text{h}}_{\text{i}}^{\text{L}}$ contains the hierarchical contextual information obtained after graph message passing. The residual fusion strategy ensures that the final representation simultaneously retains local appearance evidence and global contextual dependencies.

#### 3.4.3. Multi-Task Prediction.

Based on the final node representation ${\text{H}}_{\text{i}}$, HA-GNN performs candidate verification, bounding box refinement, and category classification through three parallel prediction heads. These tasks are jointly optimized so that the learned node representation can simultaneously support object existence judgment, localization correction, and semantic discrimination.

Existence Score Head. The existence score head estimates whether a candidate proposal corresponds to a real object. It consists of a fully connected layer followed by a sigmoid activation:


$$\begin{array}{c} s_{i}=\sigma \left(W_{\text{exist}}H_{i}+b_{\text{exist}}\right) \end{array}$$ (23)

where ${\text{s}}_{\text{i}}\in [\text{0},\text{1}]$ denotes the probability that candidate ${\text{B}}_{\text{i}}$ is a valid object proposal. This branch is used to suppress background candidates and retain reliable object hypotheses.

Bounding Box Regression Head. The bounding box regression head predicts the coordinate offsets for refining the original candidate box:


$$\begin{array}{c} \Delta B_{i}=\text{tanh}\left(W_{\text{box}}H_{i}+b_{\text{box}}\right) \end{array}$$ (24)

where $\Delta {\text{B}}_{\text{i}}=\left(\Delta {\text{x}}_{\text{i}},\Delta {\text{y}}_{\text{i}},\Delta {\text{w}}_{\text{i}},\Delta {\text{h}}_{\text{i}}\right)$ represents the normalized offsets of the box center, width, and height. The refined bounding box is obtained by applying $\Delta {\text{B}}_{\text{i}}$ to the original proposal ${\text{B}}_{\text{i}}$. This branch improves localization accuracy by correcting the spatial deviation of coarse candidate boxes.

Classification Enhancement Head. The classification enhancement head predicts the category distribution of each candidate:


$$\begin{array}{c} p_{i}=\text{Softmax}\left(W_{\text{cls}}H_{i}+b_{\text{cls}}\right) \end{array}$$ (25)

where ${\text{p}}_{\text{i}}\in R^{\text{C}}$ denotes the class probability vector over C object categories. Compared with independent classification based only on ${\text{F}}_{\text{i}}$, this branch benefits from graph-enhanced contextual representations, thereby improving classification robustness for small and low-light objects.

#### 3.4.4. Training objective.

The HA-GNN module is trained with a multi-task loss that jointly supervises candidate existence prediction, bounding box regression, and category classification. The overall loss function is defined as:


$$\begin{array}{c} L_{\text{HA}-\text{GNN}}=L_{\text{exist}}+\beta_{\text{box}}L_{\text{box}}+\beta_{\text{cls}}L_{\text{cls}} \end{array}$$ (26)

where $L_{\text{exist}}$ denotes the binary cross-entropy loss for object existence verification, $L_{\text{box}}$ denotes the bounding box regression loss, and $L_{\text{cls}}$ denotes the classification loss. The coefficients $\beta_{\text{box}}$ and $\beta_{\text{cls}}$ are used to balance the contributions of localization refinement and semantic classification.

The existence loss is formulated as:


$$\begin{array}{c} L_{exist}=-\frac{\text{1}}{N}\sum_{i=\text{1}}^{N}\left[y_{i}\;\text{log}(s_{i})+(\text{1}-y_{i})\;\text{log}(\text{1}-s_{i})\right] \end{array}$$ (27)

where ${\text{y}}_{\text{i}}\in \left\{\text{0},\text{1}\right\}$ indicates whether candidate ${\text{B}}_{\text{i}}$ is assigned to a ground-truth object.

For positive candidates, the bounding box regression loss is defined as:


$$\begin{array}{c} L_{box}=\frac{\text{1}}{N_{+}}\sum_{i=\text{1}}^{N_{+}}{Smooth}_{L_{\text{1}}}(\Delta B_{i}-\Delta B_{i}^{*}), \end{array}$$ (28)

where ${\text{N}}_{+}$ denotes the number of positive candidates and $\Delta {\text{B}}_{\text{i}}^{*}$ denotes the ground-truth regression target.


$$\begin{array}{c} L_{cls}=-\frac{\text{1}}{N_{+}}\sum_{i=\text{1}}^{N_{+}}\sum_{c=\text{1}}^{C}y_{ic}^{*}\;\text{log}\left(p_{ic}\right)y_{ic}^{*} \end{array}$$ (29)

The classification loss is calculated as:

where ${\text{y}}_{\text{ic}}^{*}$ is the one-hot class label of the positive candidate and ${\text{p}}_{\text{ic}}$ denotes the predicted probability of class c.

Through the joint optimization of these three objectives, HA-GNN learns discriminative and context-aware node representations. The existence branch filters unreliable candidates, the regression branch improves localization precision, and the classification branch enhances semantic recognition. Therefore, the proposed HA-GNN effectively converts a noisy over-complete candidate set into a refined set of high-confidence detections by exploiting both local visual features and global contextual relationships.

## 4. Experiments and analysis

This section evaluates the proposed two-stage RGB-T detection framework from both quantitative and qualitative perspectives. The evaluation is designed to comprehensively examine the overall detection performance, the effectiveness of the proposed modules, the robustness to challenging visual conditions, and the computational feasibility of the framework. Three public multi-spectral datasets, namely R-LiViT, KAIST, and FLIR, are adopted to cover diverse detection scenarios, including dense small-object scenes, day-night illumination variations, and vehicle-mounted RGB-thermal perception environments.

The remainder of this section is organized as follows. Section 3.1 describes the datasets, implementation settings, and evaluation metrics. Section 3.2 compares the proposed method with representative state-of-the-art detectors. Section 3.3 investigates the contribution of each core component through ablation experiments. Sections 3.4 and 3.5 further analyze the robustness under illumination variation and the detection performance across different object scales. Section 3.6 reports the inference latency and computational complexity. Finally, Section 3.7 presents qualitative results to illustrate the effectiveness and interpretability of the proposed framework. Unless otherwise stated, the complete two-stage system is denoted as HA-GNN in the following experiments.

### 4.1. Experimental settings

#### 4.1.1. Datasets.

**• R-LiViT Dataset:** The R-LiViT dataset is a high-resolution RGB-thermal benchmark containing aligned visible and thermal image pairs with a resolution of 1280×1280. Compared with conventional RGB-T detection datasets, R-LiViT contains a large number of dense small objects, complex illumination changes, and challenging traffic scenes. These characteristics make it suitable for evaluating the detection capability of multi-spectral models under severe scale degradation and low-light conditions. In this work, R-LiViT is used as the primary dataset for state-of-the-art comparison, ablation study, illumination robustness evaluation, scale analysis, efficiency analysis, and visualization analysis. The explicit rationale for selecting the R-LiViT dataset lies in its profound environmental representativeness for practical all-weather detection requirements. It seamlessly encompasses highly diverse weather conditions (such as heavy fog and rainy overcasts) and drastic illumination variations ranging from blinding daytime overexposure to near-zero-lux night scenes. Furthermore, it covers multiple critical traffic object categories (pedestrians, riders, cars, trucks, and traffic signs) across highly chaotic urban cross-sections, establishing rich scene diversity to validate our model’s resilience.

• **KAIST Dataset:** The KAIST multispectral pedestrian dataset is a widely used benchmark for RGB-T object detection. It contains approximately 95k aligned visible-thermal image pairs collected from driving scenarios. The dataset provides both daytime and nighttime subsets, which enables a systematic evaluation of detection robustness under different illumination conditions. Following common practice, the log-average miss rate, denoted as MR^−2^, is adopted as the main evaluation metric on KAIST. Lower MR^−2^ values indicate better detection performance. The selection rationale for incorporating KAIST is its structured day-night separation, which serves as an authoritative baseline to systematically clarify detection robustness under extreme illumination variations. Its subsets capture campus, street, and highway environments, providing critical scene diversity centered around robust traffic object categories.

• **FLIR Dataset:** The FLIR dataset consists of vehicle-mounted RGB-thermal image pairs with a resolution of 640×512. It contains typical driving scenes and is particularly challenging due to thermal crossover, sensor noise, and modality discrepancy between visible and infrared images. We use FLIR to further validate the generalization ability of the proposed framework in practical multi-spectral perception scenarios. The core rationale for selecting FLIR is its unique capacity to simulate sensor-level anomalies, such as thermal crossover (where objects and background share identical temperatures) and severe camera noise under adverse weather. This choice allows us to comprehensively examine the cross-modal interaction mechanism when one modality suffers from localized pattern failure, thereby matching the stringent demands of real-world all-weather driving perception.

#### 4.1.2. Implementation Details.

All experiments are implemented using PyTorch 2.7.0 on a workstation equipped with an NVIDIA GeForce RTX 5070 Ti Laptop GPU with 12 GB VRAM. The operating system is Windows 11. During training, the first-stage dual-path attention fusion module is optimized with the AdamW optimizer. The input resolution is set to 1280 × 1280 for high-resolution datasets and 640 × 640 for relatively low-resolution datasets, depending on the dataset configuration.

For the second-stage hierarchical attention graph neural network, each candidate proposal is regarded as a graph node. The number of neighboring nodes is set to K = 10, and the hidden dimension of the graph representation is set to 256. The candidate graph is dynamically constructed according to both appearance similarity and spatial-geometric proximity. The final detection result is obtained after candidate verification, bounding box refinement, and classification enhancement.

To reduce the influence of randomness, all quantitative results are averaged over three independent runs. The same training and testing protocol is used for all compared variants unless otherwise specified.

#### 4.1.3. Evaluation Metrics.

For the R-LiViT and FLIR datasets, detection performance is mainly evaluated using $\text{mA}{\text{P}}_{\text{50}}$ and $\text{mA}{\text{P}}_{\text{50}:\text{95}}$. The $\text{mA}{\text{P}}_{\text{50}}$ metric measures the mean average precision when the intersection-over-union threshold is fixed at 0.5, while $\text{mA}{\text{P}}_{\text{50}:\text{95}}$ reports the averaged precision over multiple IoU thresholds from 0.5 to 0.95. In addition, recall and F1-score are reported in the ablation study to further evaluate the candidate recovery capability and the balance between precision and recall.

For the KAIST dataset, we follow the standard evaluation protocol and report the log-average miss rate $\text{M}{\text{R}}^{-\text{2}}$. Specifically, All MR, Day MR, and Night MR are reported to measure the overall performance and the illumination-specific performance. A lower miss rate indicates better detection accuracy.

For computational efficiency, we report model parameters, inference speed, latency, and theoretical computational complexity. The inference speed is measured in frames per second (FPS), and the latency is measured in milliseconds.

### 4.2. Comparison with State-of-the-Art Methods

This subsection compares the proposed HA-GNN with representative object detection models and recent multi-modal fusion methods. The comparison is conducted on R-LiViT and KAIST to evaluate both general detection performance and all-weather RGB-T detection robustness.

#### 4.2.1. Results on R-LiViT.

**Fig 3:**
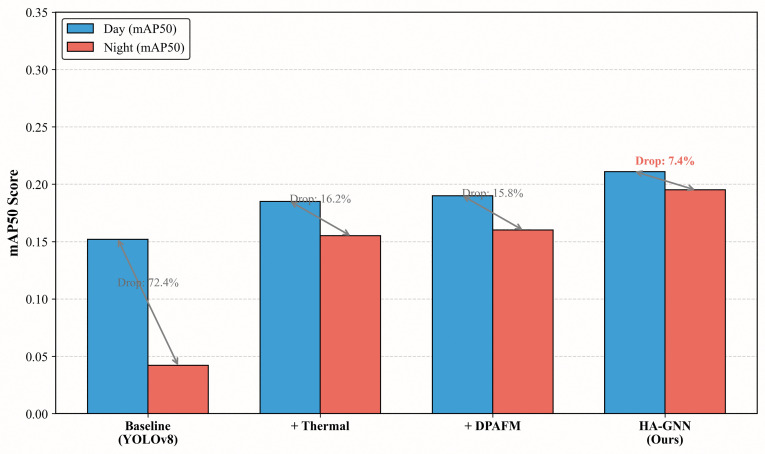
Robustness analysis under diverse illumination conditions.

Table 2 and Fig 3 reports the comparison results on the R-LiViT dataset. The proposed HA-GNN achieves the best $\text{mA}{\text{P}}_{\text{50}}$ of 22.3%, outperforming both general-purpose detectors and recent multi-modal fusion approaches.

**Table 2:** Comprehensive comparison with state-of-the-art methods on the R-LiViT dataset.

Method	Backbone	Params (M)	FPS (Hz)	mAP50 (%)
**Faster R-CNN [15]**	ResNet-50	180	20.7	15.0
**RT-DETR-L [** **48** **]**	ResNet-50	110	28.6	16.0
**SuperFusion [49]**	ResNet-50	38.5	18.2	19.5
**PMSFNet [50]**	ViT-S	22.4	24.5	20.8
**MambaSOD [12]**	Mamba	78.9	39.5	21.2
**HA-GNN (Ours)**	Fusion	82.4	27.4	22.3

Compared with Faster R-CNN and RT-DETR-L, HA-GNN achieves a substantial accuracy improvement. Specifically, Faster R-CNN obtains $\text{15}.\text{0}\%\text{mA}{\text{P}}_{\text{50}}$, while RT-DETR-L obtains $\text{16}.\text{0}\%\text{mA}{\text{P}}_{\text{50}}$. In contrast, HA-GNN improves $\text{mA}{\text{P}}_{\text{50}}$ to 22.3%, indicating that conventional detectors have limited capability in handling high-density small objects and degraded multi-spectral patterns.

Compared with multi-modal fusion methods, HA-GNN also shows clear advantages. SuperFusion achieves 19.5% $\text{mA}{\text{P}}_{\text{50}}$, while PMSFNet and MambaSOD achieve 20.8% and 21.2%, respectively. Although MambaSOD reaches the highest inference speed among the compared methods, its detection accuracy is still 1.1 percentage points lower than that of HA-GNN. This suggests that the proposed hierarchical graph reasoning mechanism is more effective in modeling candidate-level contextual dependencies, especially when local features are unreliable due to small scale, occlusion, or low illumination.

It is also worth noting that HA-GNN achieves a favorable balance between accuracy and efficiency. Although its parameter size is larger than some lightweight models, the proposed framework maintains a real-time inference speed of 27.4 FPS. Therefore, HA-GNN provides a practical trade-off between detection precision and computational cost.

#### 4.2.2. Results on KAIST.

Table 3 shows the quantitative comparison on the KAIST dataset. The proposed HA-GNN achieves the lowest All MR of 9.2%, outperforming all compared methods. In daytime scenarios, HA-GNN obtains a Day MR of 8.9%, while in nighttime scenarios, it obtains a Night MR of 9.5%. The small gap between daytime and nighttime results demonstrates that the proposed method is robust against illumination variations.

**Table 3:** Comparison with state-of-the-art RGB-T detection methods on the KAIST dataset.

Method	All MR	Day MR	Night MR
**MBNet [51]**	10.5	9.8	11.2
**CSFRNet [52]**	10.2	10.1	10.4
**TFNet [53]**	9.8	9.4	10.1
**Context-Graph [28]**	9.4	9.1	9.7
**HA-GNN (Ours)**	9.2	8.9	9.5

Compared with MBNet, HA-GNN reduces All MR from 10.5% to 9.2%. Compared with CSFRNet, the proposed method achieves a 1.0 percentage point reduction in All MR. Compared with TFNet and Context-Graph, HA-GNN still obtains lower miss rates in all evaluation settings. These results demonstrate that the combination of dual-modal feature fusion and graph-based contextual reasoning can effectively improve multi-spectral detection performance.

**Fig 4:**
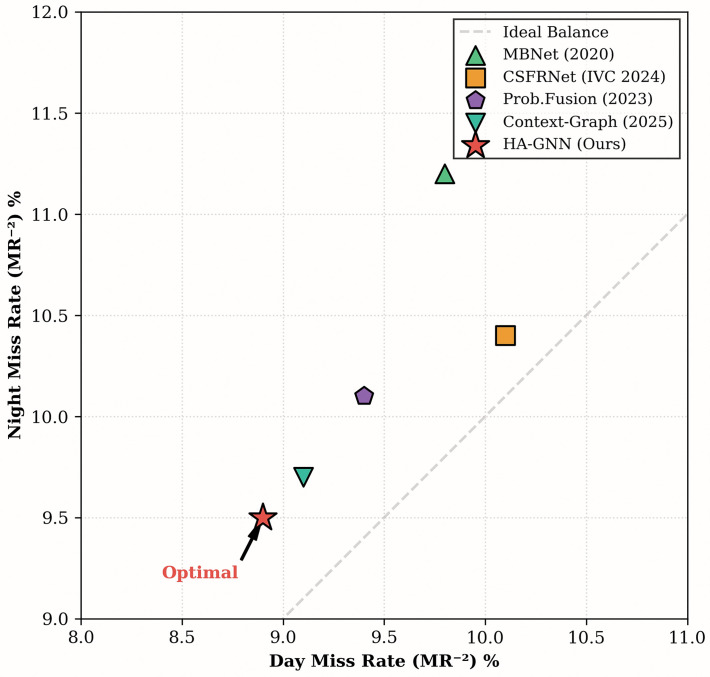
Distribution of day-night miss rates MR − 2 on the KAIST dataset.

Fig 4 further visualizes the distribution of Day MR and Night MR. The proposed HA-GNN is located closest to the origin, indicating the best overall detection performance. More importantly, HA-GNN maintains a balanced position between daytime and nighttime conditions, which confirms that the proposed framework does not overfit to a specific illumination setting. This observation supports the effectiveness of using thermal information for illumination compensation and hierarchical graph reasoning for local feature compensation.

### 4.3. Ablation Study

To verify the effectiveness of each component in the proposed framework, we conduct a stepwise ablation study on the R-LiViT dataset. The ablation results are reported in Table 4 The evaluated components include RGB input, thermal input, the Dual-Path Attention Fusion Module, and the Hierarchical Attention Graph Neural Network.

**Table 4:** Ablation study results on the R-LiViT dataset.

Model	RGB	Thermal	DPAFM	HA-GNN	Recall	$𝐦𝐀{𝐏}_{50}$	mAP50:95	F1
**Baseline**	✓				0.080	0.097	0.010	0.132
**+Thermal**	✓	✓			0.100	0.170	0.017	0.133
**+DPAFM**	✓	✓	✓		0.164	0.175	0.017	0.251
**+HA-GNN**	✓	✓	✓	✓	0.247	0.223	0.022	0.329

The RGB-only baseline obtains 9.7% mAP50,1.0%mAP50:95, and 0.080 recall. This result indicates that visible images alone are insufficient for robust detection under low-light and complex illumination conditions. After introducing the thermal modality, mAP50 increases from 9.7% to 17.0%. This significant gain demonstrates that thermal images provide complementary information when visible features are degraded.

When DPAFM is further introduced, mAP50 increases from 17.0% to 17.5%, and recall improves from 0.100 to 0.164. Although the improvement in mAP50 is moderate, the large increase in recall indicates that DPAFM is effective in recovering more potential object candidates through adaptive cross-modal fusion. This is particularly important for the subsequent graph reasoning stage because a high-recall candidate set provides a richer node set for contextual inference.

The full HA-GNN model achieves the best performance, with 22.3%mAP50,2.2%mAP50:95,0.247 recall, and 0.329 F1-score. Compared with the DPAFM-only variant, the introduction of HA-GNN improves mAP50 by 4.8 percentage points and recall by 0.083. This confirms that the second-stage graph reasoning module plays a decisive role in refining candidate proposals, suppressing false positives, and recovering weak object patterns.

**Fig 5:**
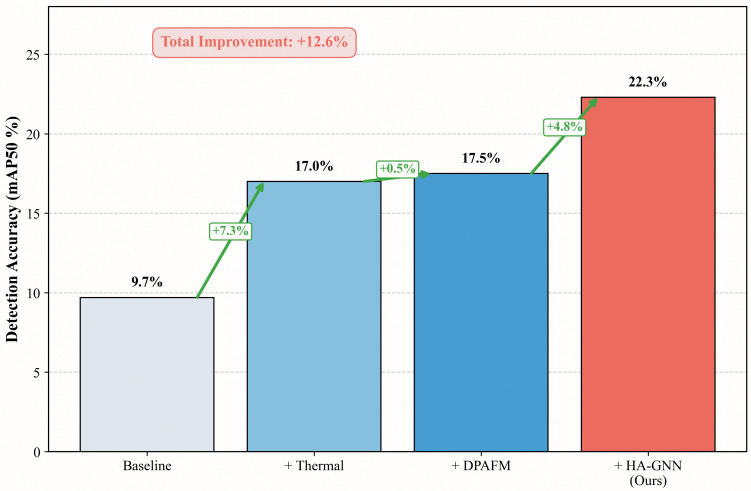
Stepwise performance gain chart of ablation experiments.

Fig 5 illustrates the stepwise performance gain of different model variants. The results show a clear progressive improvement from single-modality detection to multi-modal fusion and then to graph-based contextual reasoning. This confirms that the proposed framework is not a simple accumulation of modules, but a structured two-stage design in which the first stage enhances multi-spectral perception and the second stage performs relational verification.

### 4.4. Robustness Analysis Under Illumination Variation

Illumination variation is one of the most critical challenges in multi-spectral object detection. To evaluate the robustness of the proposed framework, we divide the R-LiViT test samples into daytime and nighttime subsets and compare the detection performance under different illumination conditions. The results are summarized in Table 5.

**Table 5:** Robustness analysis under different illumination conditions on the R-LiViT dataset.

Model	Modality	Overall $𝐦𝐀{𝐏}_{50}$	Day $𝐦𝐀{𝐏}_{50}$	Night $𝐦𝐀{𝐏}_{50}$	Drop↓
**Baseline (YOLOv8)**	RGB	0.097	0.152	0.042	−72.4%
**+Thermal**	RGB-T	0.170	0.185	0.155	−16.2%
**+DPAFM**	RGB-T	0.175	0.190	0.160	−15.8%
**HA-GNN (Ours)**	RGB-T	0.223	0.211	0.195	−7.4%

The RGB-only baseline shows a severe performance degradation from $\text{0}.\text{152}\text{mA}{\text{P}}_{\text{50}}$ during the day to $\text{0}.\text{042}\text{mA}{\text{P}}_{\text{50}}$ at night, corresponding to a 72.4% performance drop. This result confirms that visible images suffer from serious feature loss in nighttime scenes. After introducing the thermal modality, the nighttime performance improves substantially to $\text{0}.\text{155}\text{mA}{\text{P}}_{\text{50}}$, and the performance drop is reduced to 16.2%. This demonstrates the importance of thermal information for low-light detection.

The DPAFM variant further improves the stability of RGB-T fusion, reducing the performance drop to 15.8%. The full HA-GNN framework achieves the most stable performance, with $\text{0}.\text{211}\text{mA}{\text{P}}_{\text{50}}$ during the day and $\text{0}.\text{195}\text{mA}{\text{P}}_{\text{50}}$ at night. The degradation rate is only 7.4%, which is significantly lower than those of the compared variants.

These results indicate that HA-GNN improves illumination robustness from two aspects. First, DPAFM adaptively integrates visible and thermal information to reduce modality-specific degradation. Second, HA-GNN uses graph-based contextual reasoning to compensate for weak local features by aggregating information from reliable neighboring candidates. Therefore, even when the local appearance of an object is degraded at night, the model can still infer its existence from spatial layout and contextual patterns.

### 4.5. Scale-Level Detection Analysis

Small-object detection is another major challenge in the R-LiViT dataset. Due to limited pixels, weak texture, and heavy background interference, small objects are more likely to be missed or incorrectly classified. To evaluate the effectiveness of the proposed method for objects with different scales, we report the COCO-style scale-based detection results in Table 6.

**Table 6:** Performance breakdown by object scale based on COCO metrics.

Method	$𝐦𝐀{𝐏}_{50:95}$	$𝐀{𝐏}_{50}$	AP75	$𝐀{𝐏}_{S}$	$𝐀{𝐏}_{M}$	$𝐀{𝐏}_{L}$
**YOLOv8-Nano**	0.012	0.040	0.008	0.005	0.015	0.030
**RT-DETR-L[48]**	0.018	0.160	0.015	0.008	0.022	0.045
**Baseline**	0.010	0.097	0.009	0.004	0.012	0.028
**HA-GNN (Ours)**	0.022	0.223	0.025	0.015	0.035	0.052
**Improvement**	+120%	+130%	+177%	+275%	+191%	+85%

Compared with the baseline model, HA-GNN improves $\text{mA}{\text{P}}_{\text{50}:\text{95}}$ from 0.010 to 0.022, corresponding to a 120% relative improvement. For $\text{A}{\text{P}}_{\text{50}}$, HA-GNN improves the score from 0.097 to 0.223, achieving a 130% relative gain. These results show that the proposed method improves not only loose-threshold detection performance but also overall localization and classification quality across multiple IoU thresholds.

The improvement is particularly significant for small objects. The $\text{A}{\text{P}}_{\text{S}}$ score increases from 0.004 to 0.015, yielding a relative improvement of 275%. This gain is larger than the improvements for medium and large objects, which indicates that the proposed graph reasoning mechanism is especially effective for small-object recovery. This is because small objects usually have insufficient local appearance cues, while their contextual relationships with surrounding candidates remain useful. By modeling these relationships, HA-GNN can enhance weak small-object representations and reduce missed detections.

For medium and large objects, HA-GNN also achieves consistent improvements. $\text{A}{\text{P}}_{\text{M}}$ increases from 0.012 to 0.035, and $\text{A}{\text{P}}_{\text{L}}$ increases from 0.028 to 0.052. This demonstrates that the proposed framework is not limited to small-object detection but provides general benefits across different object scales.

### 4.6. Efficiency and Computational Complexity Analysis

To evaluate the practical feasibility of the proposed framework, we analyze its inference latency and computational complexity on the R-LiViT dataset. The experiment is conducted on 500 representative images covering different object densities and illumination conditions. Each image is processed 50 times on an NVIDIA RTX 5070 Ti GPU, and the average latency is recorded.

• **Comparison with state-of-the-art methods:** Table 7 presents a comprehensive efficiency comparison. MambaSOD [12] achieves the highest speed (39.5 FPS, 25.3 ms) with 78.9M parameters and 16.9G FLOPs, benefiting from its linear-complexity Mamba architecture. SuperFusion [49] and PMSFNet[50] achieve 18.2 FPS (54.9 ms) and 24.5 FPS (40.8 ms), respectively. with substantially lower parameter counts (38.5M and 22.4M) and FLOPs (55.2G and 48.4G). In comparison, our method achieves 27.4 FPS with 40.0 ms latency, 82.4M parameters, and 83.8G FLOPs.While our parameter count is higher due to the two-stage design and the multi-scale feature extraction backbone, the inference speed remains competitive and comfortably satisfies real-time requirements.Compared with SuperFusion and PMSFNet, our method achieves higher accuracy (+2.8 and +1.5 percentage points in mAP50, respectively) with moderate speed improvements over SuperFusion (27.4 vs. 18.2 FPS), demonstrating a clear advantage in balancing precision and efficiency.

**Table 7:** Detailed breakdown of inference latency and computational complexity.

Method	Params(M)	FLOPs(G)	Latency(ms)	FPS	Memory(GB)
**MambaSOD [12]**	78.9	16.9	25.3	39.5	0.32
**SuperFusion [49]**	38.5	55.2	54.9	18.2	0.14
**PMSFNet [50]**	22.4	48.4	40.8	24.5	0.08
**HA-GNN(Ours)**	82.4	83.8	40.0	27.4	0.33

• **Accuracy-efficiency trade-off:** As shown in Table 2, our method achieves the highest detection accuracy (22.3% mAP50), outperforming MambaSOD (21.2%), PMSFNet (20.8%), and SuperFusion (19.5%). Notably, compared with the fastest method MambaSOD (39.5 FPS), our method sacrifices approximately 30% inference speed (from 39.5 to 27.4 FPS) but gains 1.1 percentage points in mAP50 (from 21.2% to 22.3%). This represents a favorable accuracy-efficiency trade-off: we accept a modest speed reduction in exchange for non-trivial accuracy improvements, particularly in challenging scenarios such as small-object detection (275% improvement in AP_S) and night-time robustness (reducing the day-night performance gap from 72.4% to 7.4%). For safety-critical autonomous driving applications, such accuracy gains are arguably more valuable than marginal improvements in inference speed, as long as real-time requirements (≥25 FPS) are satisfied. Our method meets this requirement at 27.4 FPS.

**• Theoretical complexity:** The HA-GNN module has a complexity of O(N·K·d), where N is the number of candidate proposals, K is the number of neighbors (fixed to 10), and d is the hidden dimension. Thus, the graph reasoning cost scales approximately linearly with the number of candidate nodes, avoiding the quadratic complexity of fully connected graph reasoning. For memory consumption, we report the theoretical lower bound under FP32 precision (Params × 4 bytes) as a unified reference: 0.32 GB for MambaSOD, 0.14 GB for SuperFusion, 0.08 GB for PMSFNet, and 0.33 GB for our method. Actual runtime memory usage may be higher due to intermediate activations and framework overhead. Despite having the largest memory footprint among the compared methods, our framework remains feasible for deployment on modern embedded GPU platforms equipped with 8–12 GB VRAM, where the trade-off between memory consumption and detection accuracy is justified by the substantial performance gains in challenging scenarios.

### 4.7. Visualization analysis

To further demonstrate the effectiveness, interpretability, and granularity of the proposed framework, we conduct an extensive qualitative visualization on six representative scenes selected from the R-LiViT dataset. These scenes comprehensively cover several typical challenging road conditions, including distant small objects, dense occlusion, nighttime illumination degradation, overexposure, heterogeneous lighting, and combined scale-illumination degradation.

Unlike conventional object detection visualizations that only illustrate final bounding boxes, our visualization pipeline delivers a multi-level explainable diagnosis. For each of the six selected scenes, we expand the qualitative results into a structured multi-graph panel comprising six subplots (labeled as (a) to (f)):

(a) Baseline Detection: The visual bounding box outputs produced by the vanilla baseline detector.
(b) Ours Detection: The final refined bounding box predictions output by our full HA-GNN framework.
(c) Feature Similarity: A dense matrix heat map visualizing the pairwise cosine similarity among all candidate node features, showcasing how discriminative representations are shaped before graph reasoning.
(d) RGB Topology: The dynamic graph topology and structural connections established over the visible channel proposals.
(e) Thermal Topology: The spatial graph topology and relational link intensities derived across the thermal channel proposals.
(f) Contribution Analysis: A comprehensive column chart mapping the inference contribution score (quantified via graph centrality scores) for each specific candidate node, directly exposing the decision-making importance of individual objects.

White dashed boxes are embedded within the detection panels to indicate specific regions of interest (RoIs), helping associate the local visual patterns with the corresponding cross-modal fusion and graph reasoning behaviors.

**Fig 6:**
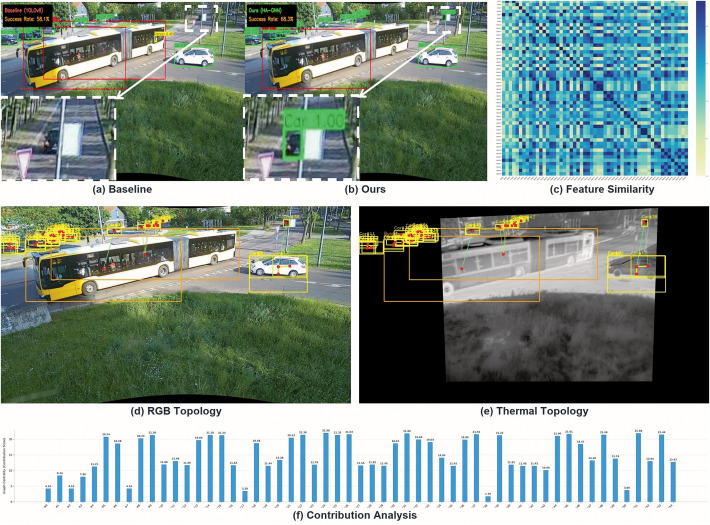
Scene 1: Scale degradation and local occlusion patterns. The underlying RGB and thermal images are sourced from the R-LiViT dataset. The detection bounding boxes, confidence scores, and category labels are generated by our proposed two-stage framework.

**Fig 7:**
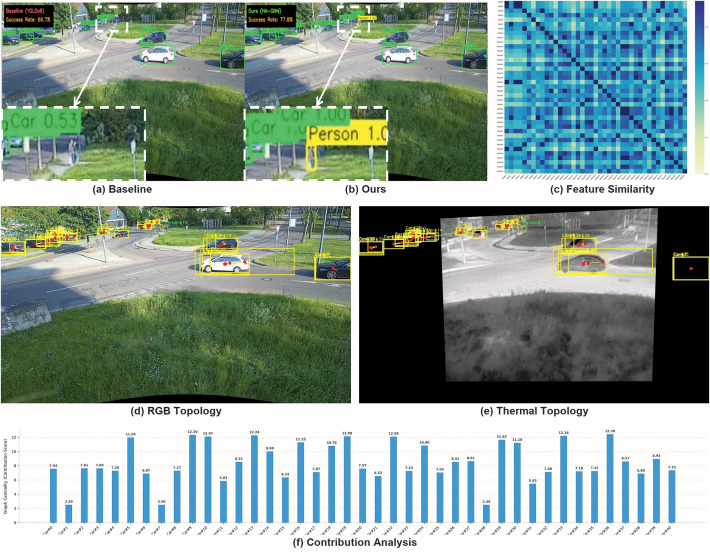
Scene 2: Scale degradation patterns under overexposure. The underlying RGB and thermal images are sourced from the R-LiViT dataset. The detection bounding boxes, confidence scores, and category labels are generated by our proposed two-stage framework.

**Fig 8:**
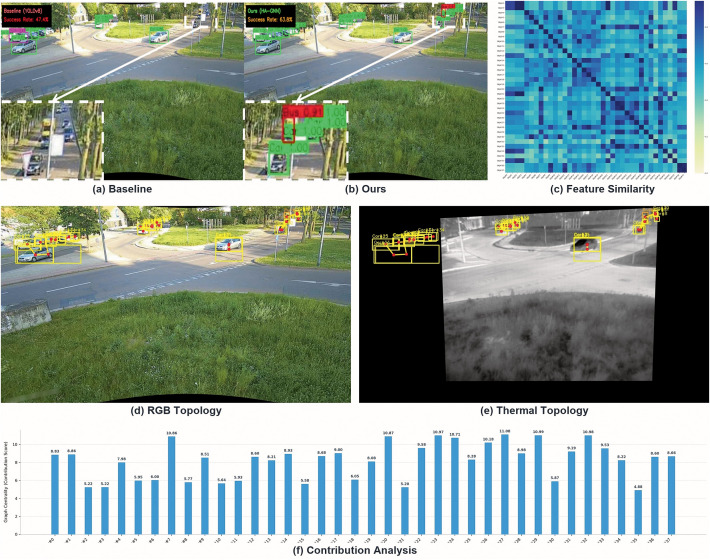
Scene 3: Scale degradation patterns in dense occlusion. The underlying RGB and thermal images are sourced from the R-LiViT dataset. The detection bounding boxes, confidence scores, and category labels are generated by our proposed two-stage framework.

**Fig 9:**
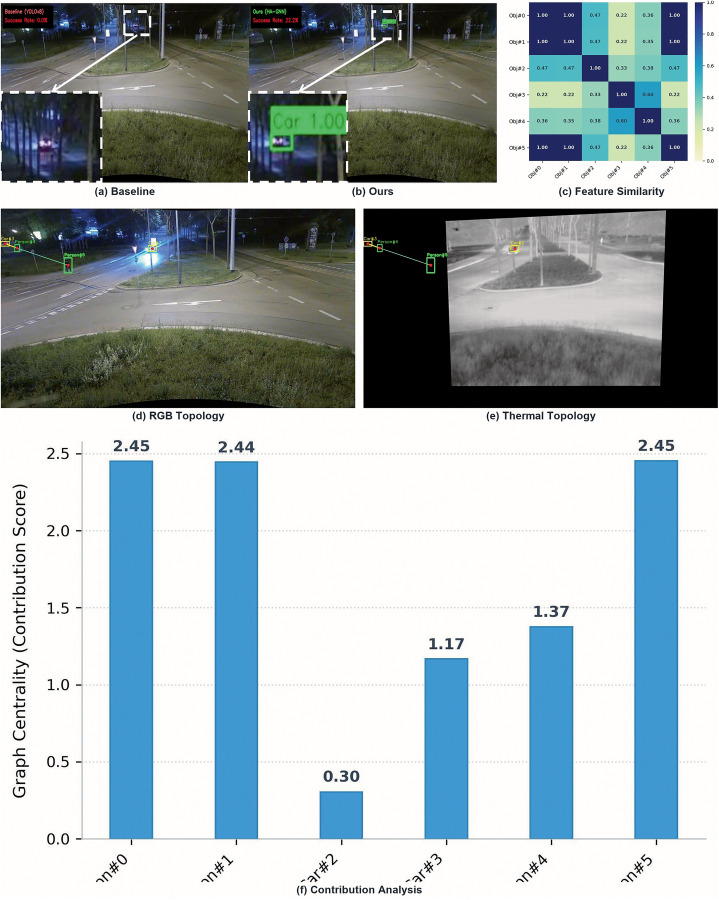
Scene 4: Multi-pattern degradation under low-light nighttime conditions. The underlying RGB and thermal images are sourced from the R-LiViT dataset. The detection bounding boxes, confidence scores, and category labels are generated by our proposed two-stage framework.

**Fig 10:**
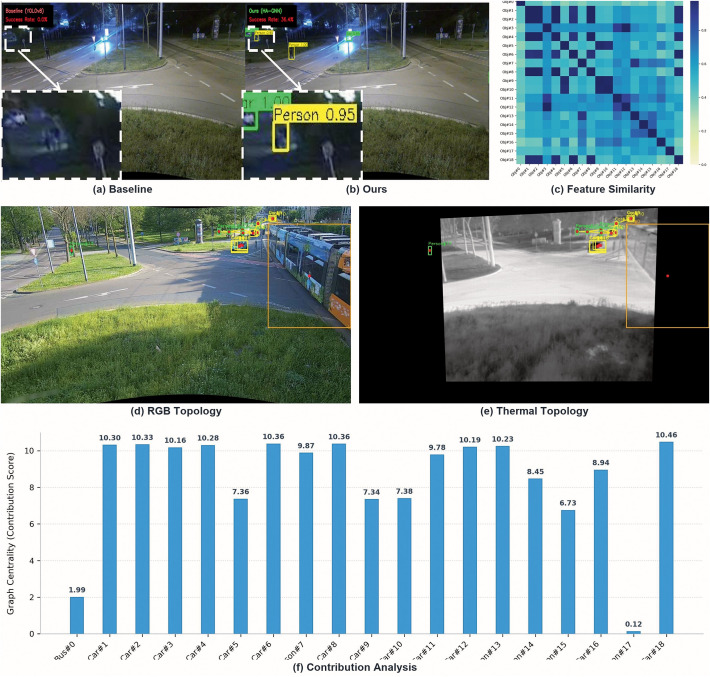
Scene 5: Composite pattern degradation under heterogeneous illumination conditions. The underlying RGB and thermal images are sourced from the R-LiViT dataset. The detection bounding boxes, confidence scores, and category labels are generated by our proposed two-stage framework.

Figs 6–10 present the highly successful qualitative results across the first five scenes. In general, the baseline detector tends to miss small, heavily occluded, or weakly illuminated objects when local visual features are severely degraded. It also produces false positives in cluttered background areas or overexposed regions. In sharp contrast, our proposed HA-GNN successfully recovers valid object candidates and delivers considerably more stable, accurate bounding boxes.

This prominent improvement is mathematically and structurally validated by subplots (c) to (f). The Node Feature Cosine Similarity Matrix (c) demonstrates that after our feature alignment, inter-class nodes maintain explicit orthogonal patterns while intra-class entities exhibit tight clusters. As depicted in the RGB and Thermal Topology Graphs (d) and (e), the hierarchical graph reasoning module actively establishes dense, high-weight relational links among visually ambiguous candidate proposals and their clear neighbors. These strong edges transfer contextual evidence across the scene layout. Consequently, as highlighted in the Contribution Analysis (f), ambiguous or weak proposals that would otherwise be discarded by the baseline receive substantial centrality reinforcement from neighboring landmark nodes (such as large vehicles or clear lane-aligned targets), lifting their confidence scores above the detection threshold.

**Fig 11:**
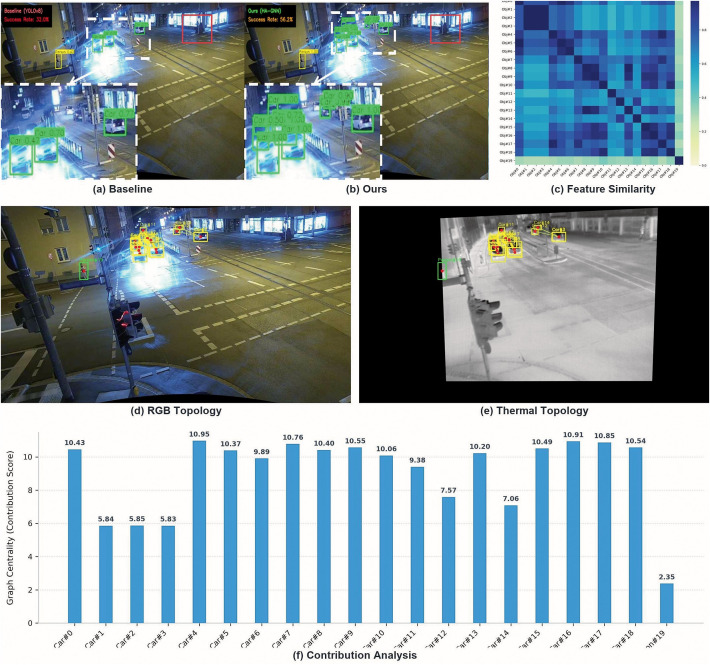
Scene 6: Dense scale patterns in overexposed environments. The underlying RGB and thermal images are sourced from the R-LiViT dataset. The detection bounding boxes, confidence scores, and category labels are generated by our proposed two-stage framework.

Despite the robust performance across most challenging driving scenarios, we present a detailed failure case analysis in Fig 11 (Scene 6) to provide an honest, objective evaluation of the system’s current boundary conditions. Scene 6 represents an extreme corner case characterized by severe combined scale-illumination degradation, where a distant pedestrian is completely missed by both the baseline and our HA-GNN framework (manually highlighted in a red solid bounding box for diagnostic purposes).

An in-depth investigation into the intermediate representations reveals the underlying cause of this failure mode:

**• Severe Sensor Degradation:** At this extreme distance under midnight illumination, the visible spectrum (RGB) suffers from absolute black-out degradation, providing zero texture or contrast for the pedestrian. Concurrently, due to severe thermal noise and long-range attenuation, the thermal sensor fails to capture the pedestrian’s heat signature, yielding a blurred, low-contrast blotch indistinguishable from background road clutter.

**• Breakdown of Graph Reasoning Primitives:** Because both input paths are heavily corrupted at the pixel level, the first-stage region proposal network (RPN) fails to generate a valid candidate node for this specific pedestrian. Since the hierarchical graph reasoning module operates exclusively on the generated candidate proposal set, it cannot reason about or recover an object that was completely omitted during the structural node initialization phase.

**• Topology Isolation:** As shown in Fig 11(c) and (f), the feature similarity and graph centrality contribution for the surrounding spatial area drop significantly, leaving the region topologically isolated without sufficient contextual support to trigger a retrospective node discovery.

This failure analysis underlines that while graph-level reasoning significantly alleviates semantic ambiguity, its robustness remains fundamentally bounded by the minimum acceptable signal-to-noise ratio (SNR) required for initial node proposal generation.

### 4.8. Discussion

The comprehensive quantitative experiments and multi-level qualitative visualizations demonstrate that the proposed two-stage framework is highly effective and robust for multi-spectral object detection under adversarial traffic conditions. The state-of-the-art comparison proves that HA-GNN achieves superior detection accuracy on the R-LiViT dataset and competitive all-weather performance on the KAIST dataset, while preserving real-time inference speed (40.0 ms latency / 27.4 FPS) suitable for edge deployment.

The detailed ablation studies and the sub-graph analyses (Feature Similarity maps and Modal Contribution charts) confirm that both the Dual-Path Adaptive Fusion Module (DPAFM) and the Hierarchical Graph Neural Network (HA-GNN) contribute positively to the final performance. In particular, HA-GNN provides the most substantial accuracy gain, which proves that candidate-level contextual reasoning and layout consistency verification are far more critical for resolving object ambiguity than merely stacking deeper pixel-level convolutional layers. The illumination robustness analysis further indicates that the thermal modality and structural graph reasoning operate in a highly complementary manner: thermal images compensate for instantaneous visible feature degradation (e.g., nighttime or overexposure), while graph reasoning compensates for weak, fragmented, or incomplete local candidate features by mining global layout contexts. The scale-level analysis further confirms that HA-GNN is particularly effective for small-object detection, where local appearance alone is often insufficient.

However, the failure case analyzed in Scene 6 exposes a critical scientific challenge common to graph-based detection paradigms: the strict dependence on proposal initialization quality. When extreme environmental factors simultaneously corrupt both the RGB and thermal signatures of a distant or minuscule object, the first-stage detector fails to instantiate a node, rendering the subsequent graph propagation powerless.

To overcome this limitation, our future research will focus on two directions:

- **Generative Cross-Modal Hallucination:** Incorporating a generative adversarial network (GAN) or a lightweight latent diffusion block within the first stage to hallucinate missing cross-modal features in severely degraded zones, thereby ensuring stable proposal generation under low SNR conditions.
- **Pixel-to-Cluster Dense Graph Fusion:** Transitioning from a pure sparse proposal-based graph to a hybrid dense-sparse graph structure, allowing pixel-level dense background contextual nodes to directly vote for and guide the discovery of highly obscured object proposals.

In summary, the proposed framework achieves a practical, state-of-the-art balance among accuracy, robustness, interpretability, and computational efficiency, establishing a solid baseline for trustworthy autonomous driving perception.

## 5. Conclusion

In this work, we have addressed the persistent challenge of object pattern degradation in complex traffic environments by proposing a two-stage framework grounded in contextual pattern verification. The framework decouples detection into over-complete candidate mining and structured reasoning through the Hierarchical Attention Graph Neural Network (HA-GNN), thereby transitioning from conventional independent classification to context-aware joint optimization. This design principle aligns with neurocomputational theories of hierarchical sensory processing and contextual integration, reflecting how biological visual systems utilize both local cues and global context to achieve robust perception.

The proposed Dual-Path Attention Fusion Module (DPAFM) adaptively integrates multi-spectral information via learnable gated attention, enabling the system to compensate for degraded RGB patterns with thermal cues through attention-driven sensory weighting. The two-stage architecture provides a systematic division of labor: the first stage emphasizes high-recall candidate generation under relaxed training constraints, while the second stage applies precise verification using graph-based relational reasoning. HA-GNN further introduces decoupled appearance and spatial attention mechanisms, facilitating contextual compensation for small or degraded objects in a manner analogous to the ventral and dorsal visual streams of human vision, which process “what” and “where” information, respectively. Extensive experiments on the R-LiViT, KAIST, and FLIR datasets validate the effectiveness of the framework, demonstrating a reduction of the day-night performance gap from 72.4% to 7.4%, a 275% improvement in small-object detection (AP_S), and more than a twofold increase in recall. Ablation studies corroborate the contribution of each module, while visualizations reveal interpretable reasoning grounded in hierarchical relational inference.

Despite its strengths, including conceptual clarity, a systematic design, and robust performance across diverse scenarios, the framework exhibits certain limitations. The current step-wise training strategy is less optimal compared with end-to-end learning paradigms. The reliance on thermal modality under extreme environmental conditions, such as heavy fog, rain, and snowfall, remains unexplored. The fixed graph construction with K = 10 neighbors may under-connect or over-connect nodes in highly crowded scenes. Moreover, the computational overhead, with an inference speed of 27.4 FPS on a laptop GPU, may present challenges for deployment in resource-constrained edge environments. Nonetheless, the general “mining + reasoning” paradigm offers broad applicability to other vision tasks involving pattern degradation, and HA-GNN serves as a versatile verification module compatible with any over-complete proposal generator. The robustness and scale analyses further provide insights into model generalization under challenging conditions, demonstrating potential for real-time deployment in autonomous vehicles and traffic surveillance when combined with pruning and knowledge distillation techniques.

Looking forward, several promising directions warrant further investigation. Extending the framework to incorporate additional sensing modalities, such as LiDAR, hyperspectral, or event cameras, could provide richer complementary information for robust perception under diverse environmental conditions. Improving robustness under extreme environmental conditions—including heavy fog, rain, snow, and thermal crossover—requires adaptive modality selection and uncertainty-aware fusion mechanisms. Reducing computational costs for real-time deployment on edge devices can be achieved through model pruning, quantization, knowledge distillation, and efficient graph sampling strategies. Enhancing interpretability by visualizing graph attention weights and reasoning paths would increase model transparency and facilitate debugging in safety-critical applications. Finally, developing foundation-model-based multimodal perception frameworks, leveraging large-scale pre-trained models (e.g., SAM, CLIP, GPT-4V) with prompt tuning or adapter modules, offers a promising direction to further boost generalization and few-shot adaptability. Collectively, these extensions underscore the potential of HA-GNN to advance robust, interpretable, and generalizable detection in complex multi-modal settings.
